# Exploring the Molecular Complexity of Medulloblastoma: Implications for Diagnosis and Treatment

**DOI:** 10.3390/diagnostics13142398

**Published:** 2023-07-18

**Authors:** Julian S. Rechberger, Stephanie A. Toll, Wouter J. F. Vanbilloen, David J. Daniels, Soumen Khatua

**Affiliations:** 1 Department of Neurologic Surgery, Mayo Clinic, Rochester, MN 55905, USA; rechberger.julian@mayo.edu (J.S.R.); daniels.david@mayo.edu (D.J.D.); 2 Department of Molecular Pharmacology and Experimental Therapeutics, Mayo Clinic, Rochester, MN 55905, USA; 3 Department of Pediatrics, Division of Hematology/Oncology, Children’s Hospital of Michigan, Detroit, MI 48201, USA; 4 Department of Neurology, Elisabeth-Tweesteden Hospital, 5022 Tilburg, The Netherlands; 5 Department of Pediatric Hematology/Oncology, Section of Neuro-Oncology, Mayo Clinic, Rochester, MN 55905, USA

**Keywords:** medulloblastoma, molecular subtypes, diagnosis, epigenetic machinery, therapeutic resistance, chemotherapy, radiation therapy, targeted therapy, immunotherapy, adoptive cell therapy

## Abstract

Medulloblastoma is the most common malignant brain tumor in children. Over the last few decades, significant progress has been made in revealing the key molecular underpinnings of this disease, leading to the identification of distinct molecular subgroups with different clinical outcomes. In this review, we provide an update on the molecular landscape of medulloblastoma and treatment strategies. We discuss the four main molecular subgroups (WNT-activated, SHH-activated, and non-WNT/non-SHH groups 3 and 4), highlighting the key genetic alterations and signaling pathways associated with each entity. Furthermore, we explore the emerging role of epigenetic regulation in medulloblastoma and the mechanism of resistance to therapy. We also delve into the latest developments in targeted therapies and immunotherapies. Continuing collaborative efforts are needed to further unravel the complex molecular mechanisms and profile optimal treatment for this devastating disease.

## 1. Introduction

Medulloblastoma is a malignant pediatric brain tumor with vastly diverse clinical outcomes based on age, the presence of residual or metastatic disease, and histopathological and molecular classification. The 2016 World Health Organization (WHO) classification system incorporated molecular entities into their diagnoses, stratifying medulloblastoma into four major subgroups: WNT-activated, sonic hedgehog (SHH), and non-WNT/non-SHH groups 3 and 4. SHH was further divided according to tumor protein 53 (TP53) status, as these tumors have markedly different clinicopathological characteristics (Table 1). In 2021, this classification was further stratified, with the four major subgroups further delineated according to methylation profiling and pathway activation [1,2,3]. These subgroups not only demonstrate the profound genetic heterogeneity among this tumor type but are imperative to profile molecularly driven treatment strategies and outcomes.

These divergent clinical outcomes, along with clearly different clustering of tumors (even within the principal subgrouping) have led to the current 2021 WHO classification system [2]. Like the 2016 classification, the WNT-activated group is present, but SHH-activated tumors (TP53 wild type) are now divided among four subgroups discovered in DNA methylation or transcriptome profiling [4,5,6]. The SHH-activated TP53 mutant is considered a separate molecular entity in the WHO classification system but falls within one of the four subgroups of TP53 wild type tumors in the more granular classification systems [4,5,6]. These subgroups not only differ in their demographic findings but in their clinical features and cytogenetic findings as well. The same holds true for non-WNT/non-SHH group 3 and 4 tumors. These tumors are now divided into a spectrum of eight new subgroups, with subgroup 1 primarily representing the previous group 3 and subgroup 8 primarily representing the previous group 4 tumors [4,6]. These novel groups provide ample opportunities to tailor care in these patients, which will hopefully result in uniformly improved outcomes among this difficult-to-treat population. The clinicopathologic and molecular (Table 2) differences among these new subgroups as well as the current and historical clinical trials incorporating them are discussed below.

## 2. Epigenetic Machinery in Medulloblastoma

**Figure 1:**
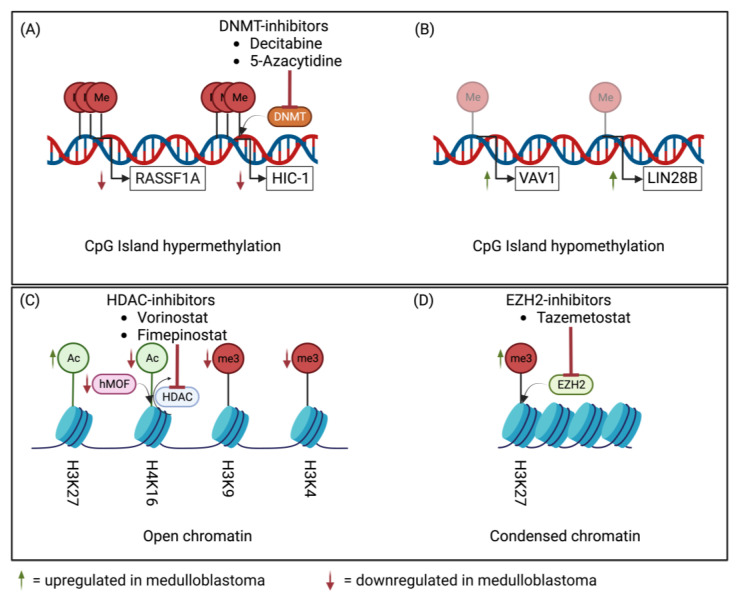
Epigenetic regulation mechanisms in medulloblastoma. (**A**,**B**) CpG island methylation aberrations result in gene silencing of tumor suppressor genes and activation of oncogenes promoting oncogenesis and tumor progression. (**A**) CpG Island hypermethylation in tumor suppressor genes RASSF1A and HIC-1, resulting in tumorigenesis. DNMT inhibitors including decitabine and 5-azacytidine inhibit this methylation. (**B**) CpG Island hypomethylation in oncogene VAV1 and LIN28B downregulating a tumor suppressor gene family and promoting tumorigenesis. (**C**,**D**) Histone modifications are a form of chromatin dysregulation that results in alterations of gene functionality. HDAC inhibitors (vorinostat and fimepinostat) and EZH2 inhibitors (tazemetostat) act on these, thereby decreasing tumorigenesis. (**C**) Acetylation of H3K27 is upregulated in medulloblastoma, permitting transcription factors that result in tumor development and propagation. However, loss of H4K16 acetylation occurs due to decreased hMOF (a histone acetyltransferase). Hypomethylation of H3K9 and H3K4 results in unchecked oncogenes that further tumor survival. (**D**) Increased H3K27me3 is seen in both group 3 and group 4 medulloblastoma in a subgroup-specific manner. Abbreviations: DNMT, DNA methyltransferase; HDAC, histone deacetylase; hMOF, human males absent on the first; EZH2, enhancer of zeste homolog 2; Me, methylation; Me3, trimethylation; Ac, acetylation. Created with BioRender.com (accessed on 1 May 2023).

The landscape of medulloblastoma is changing, and the importance of epigenetic dysregulation is becoming all the more apparent. Epigenetics, or changes in cell function not related to DNA sequences, is known to play a critical role in cancer development and progression, but only more recently has its importance become known in pediatric brain tumors. In medulloblastoma, several epigenetic aberrations have been implicated in the pathogenesis of the disease, predominantly in the form of DNA methylation and histone modification (Figure 1) [7,8]. It has been postulated that each subgroup has different alterations that drive tumorigenesis, with epigenetic changes occurring across all subgroups. In fact, more than 30% of medulloblastoma samples contain modifications of genes that encode epigenetic regulators, and while some of these overlap, each epigenetic signature is unique, providing insight into the drivers of each disease subgroup [9].

DNA methylation has been implicated in several tumors, including medulloblastoma. The most common form of this is within the context of CpG islands, which are frequently found near the promoter region of genes. Methylation of these islands results in permanent gene silencing with the possibility of silencing multiple genes with a single change [10,11]. Typically, these silenced genes are tumor suppressor genes, which then enable the tumor to grow unopposed. This is of clinical importance, as these changes carry prognostic value, particularly in medulloblastoma [12]. These methylation profiles are so profound that they have been used to further stratify the previously designated subgroups into both molecularly and clinically distinct entities as outlined by the 2021 WHO classification system [2,6].

Several tumor suppressor genes have been shown to be inactivated through hypermethylation of CpG islands. RASSF1A is a tumor suppressor gene located on chromosome 3 that has been shown to be almost ubiquitously hypermethylated in medulloblastoma cell lines and primary tumors. This hypermethylation results in irreversible biallelic inactivation of the RASSF1A gene, contributing to tumorigenesis [13]. In a study by Lusher et al., cell lines that were hypermethylated had RASSF1A expression restored after treatment with 5-aza-2′-deoxycytidine or decitabine (DNA methyltransferase inhibitor), highlighting the clinical implications of restoring epigenetic regulation in these tumors [13]. Additional methylation-related apoptosis has also been demonstrated in other studies, including the loss of caspase-8 mRNA expression. Similarly to RASSF1A, this expression can be restored with a DNA methyltransferase inhibitor (decitabine) [14]. Another example of CpG island methylation is the hypermethylation of HIC-1 (hypermethylated in cancer-1). This gene is a potent tumor suppressor gene whose hypermethylation has been established in multiple different tumor types and has been correlated with poor overall survival in medulloblastoma [15]. ZIC2 has also been correlated with poor overall survival. This gene has multiple functions, including interaction with proteins in the SHH pathway as well as that of beta-catenin inactivation, both of clinical and diagnostic importance in medulloblastoma [16].

CpG island hypomethylation has also been revealed in medulloblastoma [7]. In the SHH subgroup, VAV1 is an oncogene that is hypomethylated, leading to an increase in expression and therefore tumorigenic potential [17]. VAV1 hypomethylation is a frequent aberration among this subgroup at upwards of 70% but is rarely shown in other molecular subgroups. Like those genes above, its overexpression is associated with poorer clinical outcomes [17]. S100A4, although not containing a promoter-associated CpG island, is epigenetically regulated through CpG sites. The hypomethylation of this gene is pro-tumorigenic, resulting in increased expression in medulloblastoma [18]. In group 3 and 4 medulloblastoma, LIN28B hypomethylation downregulates a tumor suppressor gene family, as it results in a novel promoter [19].

In addition to methylation aberrations in medulloblastoma, histone modifications are also prevalent and of clinical significance. These modifications lead to changes that impact transcription, and mechanisms include acetylation, phosphorylation, methylation, and ubiquitination. One prominent example is alterations in H3K9 (histone 3, lysine 9), which is imperative to stem cell maturation and has been implicated in several cancers, including medulloblastoma. One study by Northcott et al. found that approximately 40% of medulloblastoma samples had H3K9me3 lower than controls [20]. Single copy number aberrations (SCNAs) have also been identified that impact this specific chromatin modification, including EHMT1, SMYD4, JMJD2C, MYST3, L3MBTL3, and SCML2 [9,21].

Chromatin dysregulation is not limited to H3K9 but also includes H3K4 and H3K27 methylation. The methyltransferases involved are mutated in select medulloblastoma samples irrespective of the subgroup [9]. Within groups 3 and 4, modifiers of H3K27 methylation have been described as potentiating tumor growth and proliferation. In this study, these subgroups were defined by high levels of EZH2 expression as demonstrated by H3K27me3 and impaired H3K4 methylation [22]. This methylation has been postulated to mirror the oncogenic actions that have been shown in other cancers that accelerate tumor development and propagation [20].

Histone acetylation and deacetylation are also prevalent in medulloblastoma across subgroups, in some cases correlating with prognosis [23]. Among those mutated are genes encoding for CBP and p300 (CREBBP and EP300, respectively), which acetylate H3K27 [9]. Interestingly, this alteration is disproportionally increased in SHH medulloblastoma and present in almost 19% of the subgroup [24]. hMOF is a histone acetylase for H4K16, the downregulation of which is linked to poor overall survival [23]. These aberrations are clinically relevant, and as such, HDAC inhibitors are being evaluated within the context of clinical trials in medulloblastoma.

In addition to histone modifications and methylation, microRNA (miRNA) and mRNA are now emerging as a component of tumor pathogenesis, particularly in medulloblastoma. MicroRNAs are small non-coding RNAs that downregulate gene expression, leading to mRNA cleavage or translational and transcriptional repression [25]. Clustering analysis in medulloblastoma demonstrates that primary specimens can be classified into different subgroups according to miRNA expression [25]. Both downregulation and upregulation have been reported to act on targets within the SHH pathway, the MYCN and MYC pathways, GLI1, SMO, and CDK6, among others [26]. Interestingly, miRNA expression has not only been correlated with the subgroup but also with average-risk versus high-risk classification. In one study by Ferretti et al., lower expression of two miRNAs (miR-31 and miR-153) was found in high-risk versus average-risk tumors [27]. While both upregulation and downregulation have been demonstrated, the majority of miRNAs are downregulated; suggesting that they have antiproliferative properties that are eradicated when they are no longer functioning [27]. Together, it is clear that miRNA plays a critical role in medulloblastoma development and propagation and warrants further investigation in both pathogenesis and possible clinical targets.

## 3. Current Treatment Paradigms and Novel Therapies

### 3.1. Standard-of-Care Therapy

Therapy for medulloblastoma for all subtypes is multimodal and combines surgery, chemotherapy, and (depending on age) craniospinal irradiation or autologous stem cell rescue. Specific regimens, including chemotherapeutic agents and use of irradiation, are primarily dependent on risk stratification. Historically, definitions of average risk and high risk were driven by both metastatic disease status and extent of resection; however, reliance on these definitions alone resulted in significant differences in clinical outcomes [11]. Additionally, recent study results from the Children’s Oncology Group also demonstrated this same diversity, necessitating a change in our approach to this disease entity [28]. These results, along with the molecular heterogeneity demonstrated by genetic profiling, have led to shift toward more molecularly stratified treatment regimens [11,29].

Current treatment regimens are now designated not only by age and metastatic and resection status but by medulloblastoma subtype as well. In addition, while the 2021 subgrouping provides valuable prognostic and therapeutic implications, the most recent treatment regimens are based upon the four principal subgroups from the 2016 WHO classification system. One of the most prominent examples of this is WNT-activated medulloblastoma, which has been shown to have a favorable outcome (even when metastatic) with a 5-year event-free survival (EFS) of over 90% [30,31,32]. Given this, in conjunction with previous trials revealing non-inferior outcomes with less chemotherapy, the Children’ Oncology Group is currently studying reduced irradiation and chemotherapy for this subgroup (NCT02724579). Similarly, Leary et al. published results of a high-risk medulloblastoma study (ACNS0332) in 2021 that demonstrated improved survival exclusively in the high-risk group 3 cohort when carboplatin was used in conjunction with craniospinal irradiation [28]. This study, like that of the WNT-activated study, highlighted the diversity within this disease entity. Moving forward, the therapies will likely be further segregated via definitions of high and average risk incorporating molecular findings as well as metastasis and extent of resection.

### 3.2. Targeted Therapies

**Figure 2:**
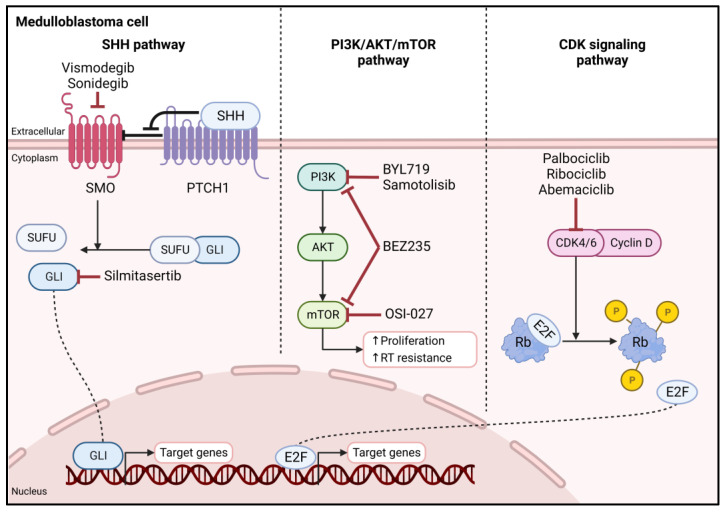
Main pathways of interest for targeted therapies in medulloblastoma. SHH pathway: in the absence of SHH, PTCH1 suppresses SMO activation. This suppression is released upon binding of SHH. Active SMO promotes GLI dissociation from SUFU, allowing for its translocation to the nucleus. The SMO inhibitors vismodegib and sonidegib and the CK2 inhibitor silmitasertib have been used to target this pathway. PI3K/AKT/mTOR pathway: PI3K/AKT/mTOR signaling pathway alterations play a crucial role in medulloblastoma. Several drugs that inhibit either PI3K, mTOR, or both are undergoing clinical evaluation. CDK signaling pathway: the cyclin D-CDK4/6 complex phosphorylates Rb, preventing binding of the E2F transcription factor. Translocation of E2F to the nucleus promotes S-phase entry and progression. Several clinical trials using CDK4/6 inhibitors in medulloblastoma are ongoing. Abbreviations: SHH, sonic hedgehog; SMO, smoothened; PTCH1, patched 1; SUFU, suppressor of fused homolog; GLI, glioma-associated oncogene homolog; CK2, casein kinase 2; PI3K, phosphatidylinositol-3-kinase; AKT, protein kinase B; mTOR, mechanistic target of rapamycin; CDK, cyclin-dependent kinase; Rb, retinoblastoma. Created with BioRender.com (accessed on 1 May 2023).

Although the last two decades have witnessed improvement in the survival rate of medulloblastoma, toxic adverse effects and concerns regarding long-term sequelae have undermined therapeutic efficacy [33]. A paradigm shift toward personalized targeted therapy for medulloblastoma that aims to reduce the side effects of standard cytotoxic agents without compromising therapeutic efficacy is now favored and pursued [34,35,36]. While personalized treatments for each medulloblastoma subgroup and subtype are still in their infancy, some driver mutations and inhibitors that target these mutations or interfere with aberrant signaling pathways have been discovered in WNT-activated and SHH-activated tumors and are undergoing extensive in vitro and in vivo testing [37]. Conversely, the biology of non-WNT/non-SHH group 3 and 4 tumors is not as well understood, which currently limits the application of novel targeted therapies to some general genetic and epigenetic targets [38]. The following section discusses some potential subgroup-specific targets for SHH and WNT medulloblastoma as well as broader targets in clinical development for all subgroups (Figure 2).

#### 3.2.1. Hedgehog Signaling Pathway

SHH-activated medulloblastoma is characterized by mutations in hedgehog (HH) target genes that lead to a constitutively active signaling pathway [37,39]. Small-molecule inhibitors that target factors implicated in HH signaling are being developed as potential therapeutics. The SMO inhibitor vismodegib (GDC-0449) is FDA approved for the treatment of SHH-dependent cancers and is in clinical testing for medulloblastoma. Tumor regression and a stable response to vismodegib were observed in patients with refractory metastatic disease [40,41]. Several clinical trials evaluating the impact of vismodegib alone or in combination with adjuvant chemotherapy were subsequently initiated (e.g., NCT01878617, NCT01601184, and NCT00822458). Results from the phase II Pediatric Brain Tumor Consortium studies PBTC-025B (NCT00939484) and PBTC-032 (NCT01239316) showed targeted efficacy of vismodegib against recurrent SHH-activated medulloblastoma, with SMO inhibitory action varying according to the genomic aberrations present in the tumor [42]. Vismodegib resistance can result from SMO mutations as well as mutations in HH genes upstream or downstream from SMO. Furthermore, it was reported that high-risk patients harboring SUFU mutations or MYCN/GLI2 amplifications did not respond to SMO inhibition but still developed growth-plate fusions because of drug exposure [43,44,45]. Sonidegib is another SMO antagonist that has been examined clinically in medulloblastoma (NCT01125800, NCT01708174). A phase I clinical trial of oral sonidegib in pediatric brain and solid tumors and a phase II study in children and adults with relapsed medulloblastoma established well-tolerable dose levels and found complete or partial responses in half of the medulloblastoma patients with activated HH signaling [46]. Preliminary reports of sonidegib in the adjuvant setting in high-risk patients or heavily pretreated patients with leptomeningeal disease further support the potential clinical benefits of this drug [47].

A viable alternative to SMO inhibition is to directly or epigenetically target the transcription factor GLI, which is a terminal effector of HH signaling that is involved in initiating the transcription of HH target genes [48,49]. Silmitasertib (CX-4945) is a potent and selective casein kinase 2 inhibitor with the ability to block GLI. This drug is presently in phase I/II clinical testing (NCT03904862) for patients with recurrent or CDK4/6 pathway relapsed SHH-activated medulloblastoma [50].

#### 3.2.2. PI3K/AKT/mTOR Pathway

Alterations of the PI3K/AKT/mTOR signaling pathway are known to play a crucial role in medulloblastoma [51,52]. Extensive preclinical research has demonstrated that drugs targeting PI3K and its downstream signaling possess radiosensitizing effects and are beneficial alone or in conjunction with adjuvant cytotoxic chemotherapy [51,53,54]. Even more anti-tumor effects were observed in vitro and in vivo with the combination of PI3K (BYL719) and mTOR (OSI-027) inhibitors as compared to either drug alone [55]. The PI3K inhibitor samotolisib (LY3023414) is being tested in two ongoing clinical trials (NCT03213678 and NCT03155620) in pediatric patients with recurrent medulloblastoma [56]. Recent studies have identified GLI as a potential target for concomitant PI3K and mTOR inhibition; as a result, combined targeting of PI3K/AKT/mTOR and HH signaling may also be effective in the treatment of medulloblastoma [55]. BEZ235, a dual PI3K-mTOR inhibitor, in combination with the HH inhibitor vismodegib significantly enhanced cisplatin-mediated cytotoxicity while preferentially suppressing MYC-amplified medulloblastoma cell growth and survival [57]. Furthermore, this drug combination delayed tumor growth and prolonged survival in a xenograft rodent model of medulloblastoma with MYC amplification.

#### 3.2.3. CDK Signaling Pathway

The CDK4/6-INK4-Rb pathway is commonly dysregulated in multiple cancers, and selective inhibition of CDK4/6 potently arrests the cell cycle of tumor cells while sparing normal cells [58,59,60]. In medulloblastoma, this pathway was identified as drug-able for all non-WNT-activated subgroups [61]. While three selective CDK4/6 inhibitors (abemaciclib, palbociclib, and ribociclib) are FDA approved for the treatment of estrogen receptor-positive metastatic breast cancer, experimental data indicate that the addition of these drugs might improve efficacy and overcome de novo or acquired treatment resistance to established therapeutic regimens in medulloblastoma [62,63]. Several clinical trials of CDK4/6 inhibitors alone or in combination with cytotoxic chemotherapy or targeted agents are ongoing. Two studies are investigating the treatment of CDK4/6 inhibitors, including palbociclib, ribociclib, or abemaciclib, in combination with temozolomide, irinotecan, topotecan, cyclophosphamide, or dinutuximab in pediatric patients with relapsed/refractory solid tumors, including medulloblastoma (NCT03709680 and NCT04238819). Another study is enrolling children and young adults with recurrent brain cancer (including group 3/group 4, WNT/SHH-activated, and SHH-activated tumors) for treatment with ribociclib in combination with gemcitabine, trametinib, or sonidegib (NCT03434262). The phase II Pediatric MATCH trial is studying the efficacy of palbociclib in patients with Rb-positive tumors (NCT03526250). A study of abemaciclib is now recruiting children and adolescents with relapsed, refractory, or progressive malignant brain tumors and solid tumors (NCT02644460).

#### 3.2.4. Epigenetic Deregulation

In the past decade, numerous studies have revealed the key role of epigenetic dysregulation in subgroup-specific tumorigenesis of medulloblastoma [9,11,64]. Research corroborating the importance of epigenetics in medulloblastoma initiation and progression has facilitated the clinical development of novel therapeutic opportunities (Figure 1) [36,37]. A Children’s Oncology Group Phase I Consortium study reported the safety and tolerability of suberanilohydroxamic acid (SAHA)/vorinostat given in combination with temozolomide in children with refractory or recurrent CNS malignancies (NCT1076530). Three patients had stable disease and one patient had a partial response, according to the published results [65,66]. Children with recurrent or refractory solid tumors, including CNS malignancies, were enrolled in a study combining vorinostat with the proteasome inhibitor bortezomib, but the findings have not yet been published (NCT01132911). The feasibility of combining vorinostat and isotretinoin was also explored in a study of 33 participants with embryonal tumors of the CNS, including medulloblastoma patients (NCT00867178). A phase I trial (PNOC016) is presently recruiting participants to examine the effects of the HDAC/PI3K inhibitor fimepinostat on brain tumors in children and young adults (NCT03893487). The EZH2 inhibitor tazemetostat is under investigation in a phase II trial of several CNS tumors, including relapsed or progressive medulloblastoma (NCT03213665). Finally, a Pediatric Brain Tumor Consortium phase I/II study is evaluating the small molecule inhibitor of casein kinase II (CK2) silmitasertib (CX-4945) in children with recurrent SHH-activated medulloblastoma (NCT03904862). The results of these studies are eagerly awaited.

### 3.3. Tumor Microenvironment and Immunotherapies

**Figure 3:**
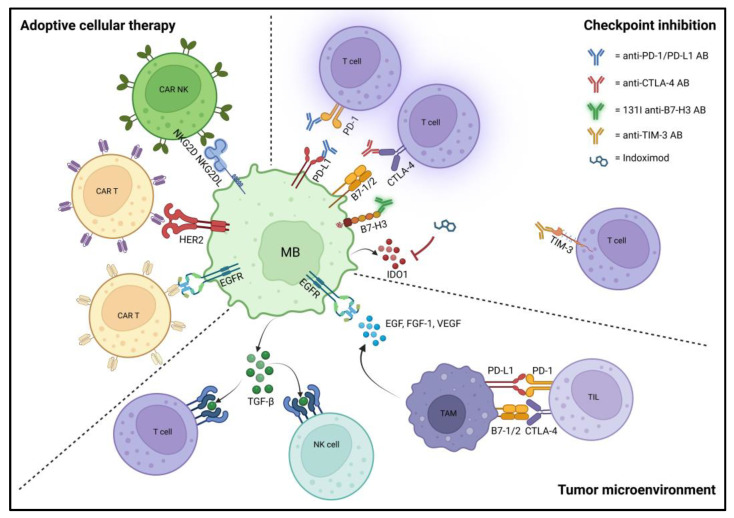
Tumor microenvironment and immunotherapies. Adoptive cellular therapy: several strategies involving engineered immune cells, including NK cells and CAR T cells targeting different receptors overexpressed in medulloblastoma, are being evaluated preclinically or are in clinical development. Checkpoint inhibition: checkpoint inhibition is increasingly investigated in medulloblastoma patients as monotherapy or in combinatorial regimens. Along with FDA-approved PD-1/PD-L1 and CTLA-4 inhibitors that are established in other tumors, newly emerging targets including B7-H3, IDO1, and TIM-3 are being explored in preclinical and clinical settings. Tumor microenvironment: medulloblastoma generally exhibits an immunosuppressive microenvironment. It is enriched in TGF-β, inhibiting NK and CD8+ T cells and driving CD4+ T cells to Treg differentiation, which in turn release more TGF-β. Furthermore, TAMs (mainly with M2 polarization) are considered the major immune cells in the medulloblastoma microenvironment, releasing several growth factors (e.g., EGF, FGF-1, and VEGF) and expressing PD-L1 and B7-1/2, thereby inhibiting T-cell function. Abbreviations: MB, medulloblastoma; CAR, chimeric antigen receptor; NK, natural killer; AB, antibody; TAM, tumor-associated macrophage; TIL, tumor-infiltrating lymphocyte; Treg, regulatory T cell. Created with BioRender.com (accessed on 1 May 2023).

There is a growing need to understand the tumor microenvironment and its role in the development and progression of medulloblastoma (Figure 3). The immune microenvironment consists of different cell types surrounding the tumor cells, including immune and non-immune cells as well as extracellularly secreted molecules, and the relationship between these can either promote or inhibit tumor growth [67]. Recent studies suggest that a lower amount of infiltrating immune cells are present in medulloblastoma than in other CNS malignancies such as glioblastoma [68,69]. Tumor-associated macrophages (TAMs) are considered the major immune cells in the medulloblastoma microenvironment and were found to be significantly more common in SHH-amplified tumors compared to other medulloblastoma subgroups [70,71]. This may be due to SHH-specific molecular signatures predictive of TAM infiltration or the high expression of monocyte chemotactic protein-1, enhancing TAM recruitment and M2 polarization [69,70,72,73]. Furthermore, glycolytic activity in response to hypoxia and acidification of the tumor microenvironment supports TAM infiltration, polarization toward an M2-like phenotype, and overexpression of programmed cell death protein ligand 1 (PD-L1) on infiltrating TAMs [74,75]. Other studies imply that the prevalence and polarization of TAMs in medulloblastoma may be age-related and associated with risk of metastatic disease and survival outcome [71,76,77,78]. Tumor-infiltrating lymphocytes (TILs) are the signaling interacting cells between TAMs and medulloblastoma cells in the tumor microenvironment. While the quantity of T cells present in medulloblastoma was not shown to be considerably higher compared to control tissues, regulatory T-cell (Treg) infiltration of the microenvironment has been described [68,69,79]. Transforming growth factor beta (TGF-β) directly inhibits CD8 T-cell activity, proliferation, and metabolism and drives differentiation of CD4 T cells to Tregs, which in turn release large amounts of TGF-β, thereby creating a feeding circuit that promotes immunosuppression [67,80,81]. Elevated levels of Tregs have been detected in the peripheral blood of patients following standard therapy, presenting a potential new strategy in the treatment of medulloblastomas [82]. However, the interaction between TAMs, TILs, other cell types, and secreted molecules in the medulloblastoma microenvironment has not been extensively studied. Further research is needed to fully elucidate the complex immune dynamics in medulloblastoma.

Due to the heterogenous nature of medulloblastoma tumors in addition to a largely non-inflammatory microenvironment with a low influx of immune cells, immunotherapeutic strategies have been challenging. The goal of immunotherapy is to stimulate and enhance the body’s natural defense system in order to slow and eventually eliminate tumor growth. Over the past 20 years, numerous immunotherapeutics have been developed for cancer treatment that have undergone intensive preclinical and clinical testing; some of these therapies have received FDA approval and are currently being used as part of innovative therapeutic regimens [83,84].

Various experimental approaches have highlighted the increasing potential for successful clinical implementation of immunotherapy (alone or in combination with alternative treatment strategies) in medulloblastoma (Figure 3) [85]. A wide spectrum of immunotherapies are in clinical development, including adoptive cellular therapy/cellular immunotherapy, immune checkpoint inhibitors, tumor vaccines, oncolytic viruses, cytokine inhibitors, and various strategies in combination with irradiation (i.e., radioimmunotherapy) [86,87,88,89,90]. Some of the major strides made in the last few years in different aspects of immunotherapy for medulloblastoma treatment are highlighted below (Table 3).

#### 3.3.1. Adoptive Cellular Therapy/Cellular Immunotherapy

In adoptive cellular therapy, host immune cells are directly isolated, modified to improve their capacity to combat cancer, and then infused back into the host to target cancer cells [91,92]. This strategy can be applied in a variety of methods, such as NK cells, engineered T-cell receptor (TCR) therapies, chimeric antigen receptor (CAR) T cells, and tumor-infiltrating lymphocytes, some of which are in clinical testing [85,86,93].

Although originally proposed in the 1980s, CAR T cells are a relatively novel and arguably one of the most promising adoptive cellular therapy strategies for the treatment of medulloblastoma [94]. Based on genetically engineering T cells of individual patients to express a receptor that specifically binds a known tumor antigen, CAR T cells have been translated clinically and have demonstrated anti-tumor activity against glioblastoma [95,96,97,98]. Human epidermal growth factor receptor 2 (HER2) is overexpressed in 38.5–63% of medulloblastomas, and multiple studies successfully evaluated HER2 CAR T in preclinical models of medulloblastoma with robust HER2 expression [99,100,101]. In vitro, HER2 CAR T cells showed HER2-dependent proliferation and secretion of IFN-γ and IL-2 [102]. They were highly potent in both medulloblastoma cell lines and autologous primary cells; furthermore, HER2-BBz-CAR T cells effectively induced tumor regression in orthotopic xenograft-bearing mice [102,103]. These findings were corroborated in non-human primates, in which intraventricular delivery of HER2 CAR T cells was feasible and safe [103]. A phase I study is currently investigating the loco-regional delivery of HER2-specific CAR T cells using autologous CD4+ and CD8+ T cells that were lentivirally transduced to express a HER2-specific CAR and truncated epidermal growth factor receptor (EGFRt) in HER2-positive recurrent/refractory CNS tumors, including medulloblastoma (NCT03500991). Similarly, autologous CD4+ and CD8+ T cells expressing an EGFR806 CAR and EGFRt are being tested in a phase I study of EGFR-positive pediatric CNS cancer patients (NCT03638167). Several other CAR T cells targeting NKG2D, B7-H3, GD2 or IL-13Rα2 showed promise preclinically and have now entered clinical development for medulloblastoma therapy (e.g., NCT05131763, NCT04185038, NCT05298995, and NCT04661384, respectively) [104,105,106].

Although in vivo studies showed promise with CAR T-cell therapy for preclinical medulloblastoma models that have led to several phase I clinical trials, to date, no phase II, III, or IV clinical trials have been registered to determine the clinical benefit of these treatments in medulloblastoma patients [84]. Compared to T cells, NK cells do not require specific tumor antigen recognition to kill tumor cells. Instead, they heavily rely on recognition of “induced self” and “missing self” antigen presentation to identify target cells, of which the most important host ligands include NKG2DLs (activatory) and MHC-I (inhibitory) [107]. The hostile microenvironment of medulloblastoma tumors is known to contain high levels of inhibitory molecules like TGF-β, which can inhibit NK cell function. Efforts have led to engineering NK cell lines that express a dominant-negative receptor (DNR) for TGF-β [108]. To date, only one phase I clinical trial (NCT02271711) of intracranial NK therapy for medulloblastoma patients has been completed [109].

#### 3.3.2. Immune Checkpoint Inhibition

Immune checkpoint inhibitors are a type of immunotherapy that block immune checkpoint proteins from binding with their partner proteins. The majority of these drugs are monoclonal antibodies that work by reducing T-cell suppression and restoring function by blocking the interaction with a particular checkpoint ligand [110]. Due to their potential to cause long-lasting tumor regression, several checkpoint inhibitors have been approved by the FDA for use in different types of cancer [111,112,113,114]. Inhibitors of cytotoxic T-lymphocyte antigen 4 (CTLA-4), PD-1, or its ligand PD-L1 are among the most prominent examples to date [115]. In addition, drugs targeting newly emerging antigens, including B7 family proteins (e.g., B7-H3), CD40/CD40L, indoleamine 2,3-dioxygenase 1 (IDO1), and mucin domain 3 (TIM-3), are demonstrating encouraging results in the treatment of brain tumors in both preclinical and clinical settings [116,117,118,119,120].

The effectiveness of immune checkpoint inhibition on preclinical models of medulloblastoma has been studied by a number of groups, who found that the different molecular subgroups may have distinctive immunological profiles that respond differently to checkpoint inhibitors [119,120,121,122]. For instance, while overall levels of PD-L1 were low, SHH-activated and low-MYC-expressing tumors had higher PD-L1 expression than non-WNT/non-SHH groups 3 and 4, according to a study comparing two cohorts of human medulloblastoma tumor samples from Children’s Hospital of Philadelphia (CHOP) and Johns Hopkins Hospital, respectively [120].

Several clinical trials of checkpoint inhibitor drugs in medulloblastoma patients are ongoing. PD-1/PD-L1 inhibitors, including nivolumab, pembrolizumab, and durvalumab, are each under investigation as monotherapy in phase I/phase II studies (e.g., NCT03173950, NCT02359565, and NCT02793466). Other studies are evaluating different combinatorial regimens such as nivolumab with and without ipilimumab (NCT03130959), indoximod in combination with ibrutinib (NCT05106296), or indoximod combined with radiochemotherapy (NCT04049669). Radiolabeled B7-H3 antibodies like 131I-omburtamab have also entered clinical testing (e.g., NCT04743661 and NCT05064306).

#### 3.3.3. Cancer Vaccination

Cancer vaccines are designed to activate an immune system that has grown tolerant to the cancer. These therapies can be categorized into multiple groups; for example whole-cell-, peptide-, DNA-, and RNA-based vaccines [123]. Tumor lysates, cells, or peptides deliver tumor antigens to antigen-presenting cells (APCs) that can then present these antigens on MHC class I and II molecules [124]. The FDA-approved sipuleucel-T vaccine consists of autologous dendritic cells that were activated with a fusion protein antigen containing a tumor-specific antigen and prostate acid phosphatase and expanded with granulocyte-macrophage colony-stimulating factor ex vivo for the treatment of prostate cancer [124,125,126]. In adult patients with newly diagnosed and recurrent glioblastoma, a phase III clinical trial recently reported clinically meaningful and statistically significant survival benefits for patients who received autologous tumor lysate-loaded dendritic cell vaccination plus the standard of care in comparison to the standard treatment alone [127,128]. This led to its evaluation in pediatric medulloblastoma. Autologous monocyte-derived dendritic cells loaded with tumor lysate for children with malignant brain tumors demonstrated that medulloblastoma responded less favorably to this therapy than high-grade gliomas and atypical/teratoid rhabdoid tumors (ATRTs) [129]. Conversely, cancer vaccines based on DNA and RNA are not restricted by HLA haplotypes, as they are transcribed and translated inside the host cells where they can then be presented to APCs to induce an immunological response [130,131,132,133,134,135]. Following proof-of-concept preclinical studies, a phase II clinical trial is currently investigating RNA-loaded autologous dendritic cells in patients with recurrent medulloblastoma and primitive neuroectodermal tumors (NCT01326104) [136]. Preliminary results were recently posted that revealed no improvement in 12-month progression-free survival in comparison to historical controls. Unfortunately, other recent clinical trials evaluating different cancer vaccine strategies in patients with medulloblastoma have been similarly unsuccessful, either lacking a robust APC response, causing severe adverse events, or failing to improve survival (NCT02332889, NCT01171469, and NCT00014573). The limited availability of viable tumor tissue for processing and vaccine generation may be partially to blame for the failure of these trials; however, the immunosuppressive nature of most medulloblastomas remains a significant hurdle for therapies that rely on the body’s own T-cell response and migration into the tumor microenvironment [86].

#### 3.3.4. Oncolytic Virotherapy

Oncolytic viral therapy is another strategy that aims to increase immune cell recognition of cancers such as medulloblastoma [93,137,138]. In general, oncolytic viruses are biotherapeutics genetically engineered to selectively infect and eradicate tumor cells, accomplishing this through distinct two processes. First, they actively kill targeted cells by propagating inside of them and bursting them. Second, through a mechanism known as epitope spreading, tumor cell lysis exposes antigens that have shed and subsequently stimulates a systemic immune response [139,140,141]. Oncolytic viruses have been studied in a variety of cancers, including brain tumors, with encouraging preclinical and clinical results [142,143,144,145,146,147]. Medulloblastoma cell lines and primary grown cells were successfully infected in vitro with oncolytic engineered myxoma-, cytomegalo-, parvo-, and poliovirus-based therapies, which further decreased cell proliferation and induced cell death [148,149,150,151]. In vivo, recombinant oncolytic myxoma, picorna, measles, and herpes simplex viruses all showed efficacy in mouse xenograft models of orthotopic and disseminated medulloblastomas, including SHH-activated and non-WNT/non-SHH group 3 and 4 tumors, following systemic, intrathecal, or intratumoral injection, respectively [148,152,153,154,155,156]. Collectively, these findings highlight the potent preclinical anti-tumor activity of oncolytic viral therapy against medulloblastoma.

A number of oncolytic viruses have now entered early-phase clinical development in patients with medulloblastoma [93,157]. Cytomegalovirus-based vaccines in combination with a preconditioning tetanus–diphtheria toxoid vaccine are being tested in the two phase I studies NCT03299309 and NCT03615404, the latter of which recently published results demonstrating the feasibility and safety of this therapy in pediatric patients with recurrent malignant glioma and medulloblastoma. A Phase 1b trial seeks to establish the safety of oncolytic poliovirus therapy when delivered intracerebrally via convection-enhanced delivery (CED) in children with various brain tumors, including medulloblastoma (NCT03043391). Another trial is employing a modified measles virus vaccine to treat children and young adults with recurrent medulloblastoma and ATRT. The vaccine is injected directly into the primary tumor or administered via lumbar puncture in the presence of metastatic disease (NCT02962167). An intravenously delivered wild-type reovirus (pelareorep) given together with subcutaneous GM-CSF (sargramostim) is used to evaluate the side effects and the best dose of this oncolytic viral therapy in young patients with high-grade brain tumors (NCT02444546). Published results from six enrolled patients demonstrate that persistent hyponatremia was the only dose-limiting toxicity, although the maximum tolerated dose was not determined [158]. Lastly, a study evaluating the safety of an oncolytic herpes simplex virus alone or in combination with a single low dose of radiation (5 Gy) is recruiting children with recurrent or progressive cerebellar brain tumors (NCT03911388).

## 4. Mechanisms of Resistance

Treatment resistance in medulloblastoma presents an ongoing challenge despite the increasing molecular understanding of medulloblastoma subtypes and oncogenic drivers. Approximately 30% of patients will relapse under the current standard of care, entailing an almost universally lethal prognosis [159]. Furthermore, newly developed targeted therapies are frequently met with development of secondary resistance, limiting clinical applications [44,160].

Recently, efforts have been made to identify transcriptional, genetic, and epigenetic drivers of treatment resistance in the current standard treatment. Taylor et al. established the role of transcription factor Y-box binding protein 1 (YB-1), showing a correlation of YB-1 overexpression with multidrug pump ATP binding cassette subfamily B member 1 (ABCB1) upregulation, inducing vincristine resistance [161]. Interestingly, YB-1 overexpression also correlated with invasiveness, metastatic potential, and MYC and mTOR pathway upregulation. Furthermore, they identified a seven-gene signature (LTBP1, MAP1A, MBNL2, LGALS1, PNRC1, DAB2, and PLAAT3) that characterized cisplatin- and vincristine-tolerant group 3 and SHH cell lines, several of which also showed increased expression in post-treatment patient samples. Other molecular mechanisms, including epigenetic alterations such as loss of H3K27me3 as a marker of resistance to irradiation and differential miRNA expression, have also been implicated in therapy resistance [162,163,164]. Increasingly, these molecular drivers are being linked to reprogramming of tumor cell metabolism, enhancing aerobic glycolysis, lipogenesis, and glutamine metabolism, which correlates with increased resilience to chemotherapy [164,165]. However, literature regarding the molecular drivers of resistance is scarce, and the interplay between these mechanisms remains to be elucidated.

On a cellular level, another mechanism of resistance in medulloblastoma is the existence of cancer stem cells (CSCs), a cell population exhibiting enhanced tumorigenesis, invasiveness, and resistance to standard chemoradiotherapy [166]. The inability to eradicate CSCs with the current standard treatment regimens allows for tumor recurrence, resulting in a tumor cell population of the same molecular subgroup but often enriched in TP3-pathway- and apoptosis-related gene alterations, resulting in therapy resistance [167,168,169,170]. A well-established CSC marker is CD133 (promenin-1), a known predictor of poor survival in medulloblastoma that is associated with resistance to both chemotherapy and radiotherapy [166,171,172,173]. Other CSC-like populations have been identified in SHH medulloblastoma mouse models expressing Nestin, Olig2, and/or Sox2 [170,174]. These populations, which resemble Nestin-expressing granule precursor cells, have been shown to be more resistant to radiotherapy by resisting p53-pathway activation [170]. Although largely quiescent pretreatment, they proliferate rapidly upon RT and/or chemotherapy, at least in part through activation of the PI3K/AKT/mTOR pathway, thereby driving tumor recurrence [55,174,175]. Recently, advancements in metabolomic strategies have allowed for the detection of CSCs according to their metabolic signature, providing novel strategies for the identification and targeting of these cells [165].

Considering that the failure of progenitor stem cells to differentiate during hindbrain maturation is thought to be the cause of medulloblastoma, CSCs are likely to coincide with the medulloblastoma cells of origin [176]. WNT signaling activation in the lower rhombic lip progenitor cells causes WNT-activated medulloblastoma, while upregulated SHH signaling in the cerebellar granule neuron precursors of the external granule cell layer leads to SHH medulloblastoma [177,178,179]. Although non-WNT/non-SHH group 3 and group 4 medulloblastoma remain the least understood, recently the unipolar brush cell progenitor in the subventricular zone of the upper rhombic lip has been put forward as the cell of origin of group 4 medulloblastoma (despite some medulloblastoma cells also showing transcriptional resemblance to granule neuron precursor cells), whereas group 3 medulloblastoma seems to arise from earlier, undifferentiated progenitor cells [176,180,181]. With different progenitor cells giving rise to medulloblastoma subgroups, different CSC populations could correlate with the distinct relapse rates and patterns [166]. Many of the characteristic pathways (e.g., the WNT pathway, SHH pathways, and MYC amplification) are related to stemness and are increasingly being evaluated as therapeutic targets, especially in SHH and group 3 tumors [33]. However, specifically targeting a single pathway often results in the development of secondary resistance through clonal selection or escape pathway upregulation, leading to a highly variable and often transient response [41,42,45]. For example, using the SMO inhibitor vismodegib, mutations in the binding pocket of SMO have been shown upon treatment, leading to an inability to exert its therapeutic effect [44]. Furthermore, patients with downstream SHH-pathway alterations such as SUFU and MYCN/GLI2 amplifications do not respond to SMO inhibition [44,45]. Moreover, while several mTOR inhibitors have been evaluated, IDO1-mediated immune escape and Mnk2-mediated eIF4E pathway activation have been shown to limit effectiveness in medulloblastoma [182,183].

Notwithstanding the progressive unraveling of the molecular underpinnings in medulloblastoma, the molecular drivers of treatment resistance remain poorly understood. However, potentially targetable mechanisms are increasingly being identified, and although targeting a single pathway almost invariably leads to resistance, further evaluation of rationally designed combination therapies could provide novel therapeutic options in treatment-resistant medulloblastoma. Furthermore, the deepened understanding of the cells of origin of the medulloblastoma subtypes has rekindled the old hypothesis that collections of atypical cells regularly detected in the postnatal nodulus could represent precursory lesions to certain medulloblastoma subtypes [184]. Hendrikse et al., in proposing the name “persistent rhombic lip” (PeRL, as opposed to the earlier “cerebellar heterotopias”), postulated that early detection and monitoring of these cells could even enable early intervention, possibly preventing group 3 or 4 medulloblastoma in the future [179].

## 5. Conclusions

Despite major advancements in cancer genomics and molecular diagnostics over the past decades, surgical resection, irradiation, and adjuvant chemotherapy generally remain the standard of care for all medulloblastoma subgroups. This has several significant shortcomings, including short- and long-term side effects. Major efforts have been made to identify subgroup-specific gene mutations and amplifications as potential targets for customized therapeutic methods, with some of them being tested in clinical trials. Immunotherapy has emerged as another promising avenue in medulloblastoma treatment, whereby adoptive cellular therapy and checkpoint inhibition are the frontrunners of a vast array of diverse immunotherapeutic strategies in preclinical and clinical testing. Understanding the tumor microenvironment of medulloblastoma and the interactions between different cell types could provide valuable insights into mechanisms of resistance and potentially lead to new targeted therapies. Furthermore, the deepened understanding of the cells of origin giving rise to distinct medulloblastoma subtypes—most likely coinciding with CSCs driving tumor recurrence—potentially allows for the development of therapies to eradicate these cells specifically or even early, presymptomatic detection and interventions. Future medulloblastoma research will profile personalized treatments for each individual patient based on molecular risk stratification of the disease with the hope of improving survival and reducing relapses.

## Figures and Tables

**Table 1:** Clinical characteristics of the principal groups of medulloblastoma.

	WNT	SHH		Non-WNT/Non-SHH	
				Group 3	Group 4
% of medulloblastoma	10	30		25	35
Gender (M:F)	1:1	1:1		2:1	3:1
Age group	Child > adult	Infant, adult > child		Infant, child	Child > infant, adult
Histology	Classic, rarely LCA	Desmoplastic/nodular> classic, MBEN, LCA		Classic, LCA	Classic, LCA
Immunoprofile	B-catenin nuc (+) YAP1/filamin A (+) GAB1 (−)	B-catenin nuc (−) YAP1/filamin A (+) GAB1 (+)		B-catenin nuc (−) YAP1/filamin A (+) GAB1 (−)	
Proposed cell of origin	Lower rhombic lip progenitor cells	CGNPs of the EGL		Undifferentiated cerebellar stem cells	Unipolar brush cells
Tumor location	Fourth ventricle; infiltrating brainstem	Cerebellar hemispheres; rarely midline		Fourth ventricle; midline	Fourth ventricle; midline
Metastasis at diagnosis	5–10%	15–20%		40–45%	35–40%
Prognosis (5-year overall survival %)	>95%	TP53-wild type: 80%	TP53 mutated (SHH-α): 40%	50%	75%

Abbreviations: LC/A, large cell/anaplastic; MBEN, medulloblastoma with extensive nodularity; CGNPs, cerebellar granule neuron precursors; EGL, external granule cell layer.

**Table 2:** Molecular characteristics and targeted therapies for the principal groups of medulloblastoma.

	WNT	SHH	Non-WNT/non-SHH	
			Group 3	Group 4
Proposed number of subtypes	2 (WNT-α and WNT-ß)	4 (SHH-α, SHH-ß, SHH-γ, and SHH-δ)	8 (Group 3/Group 4 subtypes I-VIII)	
Cytogenetics	Monosomy 6	Loss of 9q, 10q, 14q, and 17p Gain of 3q and 9p	Loss of 8q, 10q, 11q, 15q, 16q, and 17p Gain of 1q, 7, and 18 Isochromosome: 17q	Loss of 8p, 10p, 11, and 17p Gain of 4, 7q, 17, and 18q Isochromosome: 17q
Genomic abnormalities (most prevalent)	CTNNB1, DDX3X, SMARCA4, KMT2D, CREBBP, CDH1, MYC, APC, ARD1A, ARID2, and TP53	PTCH1, PALB2, BRCA2, TP53, MYCN, KMT2D, SUFU, SMO, GLI2, YAP1, IDH1, and TERT	MYC, GLI1B, GFI1, OTX2, DDX31, SMARCA4, PALB2, and BRCA2	MYCN, CDK6, SNCAIP, KDM6A, PALB2, and BRCA2
Expression signature	WNT signaling	SHH signaling	MYC signature; photoreceptor/GABAergic signature	Neuronal/glutamatergic signature
Genetic targets	PARP, EGFR, WEE-1, and ALK	PARP, EGFR, WEE-1, and ALK	PARP, EGFR, WEE-1, and ALK	PARP, EGFR, WEE-1, and ALK
Epigenetic targets	HDAC and BET/BRD	SMO, HDAC, and BET/BRD	HDAC, BET/BRD, and EZH2	HDAC, BET/BRD, EZH2, and CDK4/6

**Table 3:** Clinical trials involving immunotherapy in medulloblastoma.

Therapy Type	Title	Intervention	Patient Age	Enrollment	Phase	Status	Trial ID
Adoptive cellular therapy	HER2-specific CAR T Cell Locoregional Immunotherapy for HER2-positive Recurrent/Refractory Pediatric CNS Tumors	Biological: HER2-specific chimeric antigen receptor (CAR) T cell	1 year to 26 years	48	1	Recruiting	NCT03500991
	EGFR806-specific CAR T Cell Locoregional Immunotherapy for EGFR-positive Recurrent or Refractory Pediatric CNS Tumors	Biological: EGFR806-specific chimeric antigen receptor (CAR) T cell	1 year to 26 years	11	1	Active but not recruiting	NCT03638167
	NKG2D-based CAR T-cells Immunotherapy for Patient With r/r NKG2DL+ Solid Tumors	Biological: NKG2D-based CAR T-cells	18 years to 75 years	3	1	Recruiting	NCT05131763
	Study of B7-H3-Specific CAR T Cell Locoregional Immunotherapy for Diffuse Intrinsic Pontine Glioma/Diffuse Midline Glioma and Recurrent or Refractory Pediatric Central Nervous System Tumors	Biological: SCRI-CARB7H3(s); B7H3-specific chimeric antigen receptor (CAR) T cells	1 year to 26 years	90	1	Recruiting	NCT04185038
	GD2-CAR T Cells for Pediatric Brain Tumors	Biological: GD2-CART01 (iC9-GD2-CAR T-cells)	6 months to 30 years	54	1	Not yet recruiting	NCT05298995
	Brain Tumor-Specific Immune Cells (IL13Ralpha2-CAR T Cells) for the Treatment of Leptomeningeal Glioblastoma, Ependymoma, or Medulloblastoma	Biological: IL13Ralpha2-specific hinge-optimized 41BB-co-stimulatory CAR truncated CD19-expressing autologous T lymphocytes	18 years and older	30	1	Recruiting	NCT04661384
	Expanded Natural Killer Cell Infusion in Treating Younger Patients with Recurrent/Refractory Brain Tumors	Biological: natural killer cell therapy	0 years to 21 years	12	1	Completed	NCT02271711
Immune checkpoint inhibition	Immune Checkpoint Inhibitor Nivolumab in People with Recurrent Select Rare CNS Cancers	Drug: nivolumab	18 years to 99 years	180	2	Recruiting	NCT03173950
	Pembrolizumab in Treating Younger Patients with Recurrent, Progressive, or Refractory High-Grade Gliomas, Diffuse Intrinsic Pontine Gliomas, Hypermutated Brain Tumors, Ependymoma or Medulloblastoma	Biological: pembrolizumab	1 year to 29 years	110	1	Recruiting	NCT02359565
	Durvalumab in Pediatric and Adolescent Patients	Drug: durvalumab (MEDI4736)	1 year to 17 years	36	1	Unknown	NCT02793466
	A Study to Evaluate the Safety and Efficacy of Nivolumab Monotherapy and Nivolumab in Combination with Ipilimumab in Pediatric Participants with High Grade Primary Central Nervous System (CNS) Malignancies	Biological: nivolumab Biological: ipilimumab	6 months to 21 years	166	2	Completed	NCT03130959
	Chemo-immunotherapy Using Ibrutinib Plus Indoximod for Patients with Pediatric Brain Cancer	Drug: ibrutinib Drug: indoximod Drug: cyclophosphamide Drug: etoposide	12 years to 25 years	37	1	Recruiting	NCT05106296
	Pediatric Trial of Indoximod with Chemotherapy and Radiation for Relapsed Brain Tumors or Newly Diagnosed DIPG	Drug: indoximod Radiation: partial radiation Radiation: full-dose radiation Drug: temozolomide Drug: cyclophosphamide Drug: etoposide Drug: lomustine	3 years to 21 years	140	2	Recruiting	NCT04049669
	131I-Omburtamab, in Recurrent Medulloblastoma and Ependymoma	Drug: irinotecan Drug: temozolomide Drug: bevacizumab Drug: omburtamab I-131 Drug: liothyronine Drug: SSKI Drug: dexamethasone	Up to 21 years	62	2	Active but not recruiting	NCT04743661
	131I-omburtamab for the Treatment of Central Nervous System/Leptomeningeal Neoplasms in Children and Young Adults	Drug: 131I-omburtamab	Child, adult, and older adult	52	2/3	Available	NCT05064306
Cancer vaccination	Vaccine Immunotherapy for Recurrent Medulloblastoma and Primitive Neuroectodermal Tumor	Biological: TTRNA-xALT Biological: TTRNA-DCs	Up to 30 years	26	2	Active but not recruiting	NCT01326104
	Decitabine/Vaccine Therapy in Relapsed/Refractory Pediatric High Grade Gliomas/Medulloblastomas/CNS PNETs	Biological: vaccine (autologous dendritic cells) Drugs: decitabine and hiltonol	2 years to 25 years	1	1/2	Terminated	NCT02332889
	Vaccination With Dendritic Cells Loaded with Brain Tumor Stem Cells for Progressive Malignant Brain Tumor	Biological: dendritic cells Drug: imiquimod	Child, adult, and older adult	8	1	Completed	NCT01171469
	Chemotherapy and Vaccine Therapy Followed by Bone Marrow or Peripheral Stem Cell Transplantation and Interleukin-2 in Treating Patients with Recurrent or Refractory Brain Cancer	Biological: aldesleukin Biological: autologous tumor cell vaccine Biological: filgrastim Biological: sargramostim Biological: therapeutic autologous lymphocytes Drug: carmustine Drug: cisplatin Drug: cyclophosphamide Drug: paclitaxel	Up to 65 years	N/A	2	Completed	NCT00014573
Oncolytic virotherapy	PEP-CMV in Recurrent Medulloblastoma/Malignant Glioma	Drug: PEP-CMV	3 years to 35 years	30	1	Active but not recruiting	NCT03299309
	Cytomegalovirus (CMV) RNA-Pulsed Dendritic Cells for Pediatric Patients and Young Adults with WHO Grade IV Glioma, Recurrent Malignant Glioma, or Recurrent Medulloblastoma	Biological: CMV-DCs with GM-CSF Biological: Td (tetanus toxoid)	0 years to 35 years	11	1	Completed	NCT03615404
	Phase 1b Study PVSRIPO for Recurrent Malignant Glioma in Children	Biological: polio/rhinovirus recombinant (PVSRIPO)	12 years to 21 years	12	1	Active but not recruiting	NCT03043391
	Modified Measles Virus (MV-NIS) for Children and Young Adults with Recurrent Medulloblastoma or Recurrent ATRT	Biological: modified measles virus	12 months to 39 years	46	1	Recruiting	NCT02962167
	Wild-Type Reovirus in Combination with Sargramostim in Treating Younger Patients with High-Grade Relapsed or Refractory Brain Tumors	Biological: sargramostim Biological: Wild-type reovirus	10 years to 21 years	6	1	Active but not recruiting	NCT02444546
	HSV G207 in Children with Recurrent or Refractory Cerebellar Brain Tumors	Biological: G207	3 years to 18 years	15	1	Recruiting	NCT03911388

All data concerning the clinical trials were obtained from ClinicalTrials.gov (accessed 7 April 2023).

## Data Availability

No new data were created or analyzed in this study. Data sharing is not applicable to this article.
